# Association between regional economic status and renal recovery of dialysis-requiring acute kidney injury among critically ill patients

**DOI:** 10.1038/s41598-020-71540-7

**Published:** 2020-09-03

**Authors:** Chih-Chung Shiao, Yu-Hsing Chang, Ya-Fei Yang, En-Tzu Lin, Heng-Chih Pan, Chih-Hsiang Chang, Chun-Te Huang, Min-Tsung Kao, Tzung-Fang Chuang, Yung-Chang Chen, Wei-Chih Kan, Feng-Chi Kuo, Te-Chuan Chen, Yung-Ming Chen, Chih-Jen Wu, Hung-Hsiang Liou, Kuo-Cheng Lu, Vin-Cent Wu, Tzong-Shinn Chu, Mai-Szu Wu, Kwan-Dun Wu, Ji-Tseng Fang, Chiu-Ching Huang

**Affiliations:** 1 Division of Nephrology, Department of Internal Medicine, Camillians Saint Mary’s Hospital Luodong, Yilan, Taiwan; 2 Saint Mary’s Junior College of Medicine, Nursing and Management, Yilan, Taiwan; 3 Division of Nephrology, Department of Internal Medicine, Nan-Men Hospital, Pingtung, Taiwan; 4 Division of Nephrology, Department of Internal Medicine, China Medical University Hsinchu Hospital, Zhubei City, Hsinchu County Taiwan; 5 Division of Nephrology, Department of Internal Medicine, Lotung Poh-Ai Hospital, Luodong Township, Yilan, Taiwan; 6 Division of Nephrology, Department of Internal Medicine, Keelung Chang Gung Memorial Hospital, Keelung, Taiwan; 7 Kidney Research Center, Department of Nephrology, Linkou Chang Gung Memorial Hospital, Chang Gung University College of Medicine, Taoyuan City, Taiwan; 8 Division of Internal and Critical Care Medicine, Department of Critical Care Medicine, Taichung Veterans General Hospital, Taichung, Taiwan; 9 Division of Nephrology, Cheng-Ching Hospital, Taichung, Taiwan; 10 Division of Nephrology, Department of Internal Medicine, Nantou Hospital, Ministry of Health and Welfare, Nantou, Taiwan; 11 Division of Nephrology, Department of Internal Medicine, Chi-Mei Medical Center Yongkang, Tainan, Taiwan; 12 Division of Nephrology, Department of Internal Medicine, Mackay Memorial Hospital Taitung Branch, Taitung, Taiwan; 13 Division of Nephrology, Department of Internal Medicine, Kaohsiung Chang Gung Memorial Hospital, Kaohsiung, Taiwan; 14 Division of Nephrology, Department of Internal Medicine, National Taiwan University Hospital, 7 Chung-Shan South Road, Zhong-Zheng District, Taipei, 100 Taiwan; 15 Division of Nephrology, Department of Internal Medicine, Mackay Memorial Hospital, Taipei, Taiwan; 16 Division of Nephrology, Sin-Ren Hospital, New Taipei City, Taiwan; 17 Division of Nephrology, Department of Internal Medicine, Fu-Jen Catholic University Hospital, School of Medicine, Fu-Jen Catholic University, New Taipei City, Taiwan; 18 Division of Nephrology, Department of Internal Medicine, School of Medicine, College of Medicine, Taipei Medical University, Taipei, Taiwan; 19 Division of Nephrology, Department of Internal Medicine, China Medical University Hospital, Taichung, Taiwan

**Keywords:** Kidney, Kidney diseases, Renal replacement therapy

## Abstract

The association between regional economic status and the probability of renal recovery among patients with dialysis-requiring AKI (AKI-D) is unknown. The nationwide prospective multicenter study enrolled critically ill adult patients with AKI-D in four sampled months (October 2014, along with January, April, and July 2015) in Taiwan. The regional economic status was defined by annual disposable income per capita (ADIPC) of the cities the hospitals located. Among the 1,322 enrolled patients (67.1 ± 15.5 years, 36.2% female), 833 patients (63.1%) died, and 306 (23.1%) experienced renal recovery within 90 days following discharge. We categorized all patients into high (n = 992) and low economic status groups (n = 330) by the best cut-point of ADIPC determined by the generalized additive model plot. By using the Fine and Gray competing risk regression model with mortality as a competing risk factor, we found that the independent association between regional economic status and renal recovery persisted from model 1 (no adjustment), model 2 (adjustment to basic variables), to model 3 (adjustment to basic and clinical variables; subdistribution hazard ratio, 1.422; 95% confidence interval, 1.022–1.977; *p* = 0.037). In conclusion, high regional economic status was an independent factor for renal recovery among critically ill patients with AKI-D.

## Introduction

Acute kidney injury (AKI) is a common and complex clinical entity associated with increased medical and economic burden^1,2^. Among the critically ill patients in the intensive care unit (ICU), about 50% have AKI^3^, and 4–15% have severe AKI necessitating renal replacement therapy (RRT)^4^. The in-hospital mortality rate of patients with dialysis-requiring AKI (AKI-D) might be up to 50–80%^4^. Among these patients with AKI-D, around 13–29% are dialysis-dependent at hospital discharge^5^, and 10–30% of patients with dialysis dependence are subsequently diagnosed with end-stage renal disease (ESRD)^6,7^.

It is well known that compared to renal recovery, the non-recovery of kidney function after AKI is associated with higher mortality and economic burden^8^. Several factors, such as older age, diabetes mellitus, congestive heart failure, the severity of underlying chronic kidney disease (CKD)^9,10^, as well as the severity of AKI^11^ and the number of AKI episode^12^, were considered as risk factors for non-recovery of AKI-D. However, the association between economic status and renal recovery among patients with AKI-D is rarely illustrated.

Previous studies evaluating the association between economic status and AKI disclosed that the predominant etiologies of AKI are different in regions with different economic statuses. AKI in lower economically developed regions, comparing to that in higher economic regions, is associated with less comorbidity burden and disease severity but may be associated with even poor prognoses due to the delayed recognition and treatment^13–15^. As to the recovery rate of AKI, it was higher in less economically developed regions than in higher economic regions^13^. These findings were explained by the young age and fewer comorbidities burden in patients in less economically developed regions. Nevertheless, the definitions of AKI and kidney recovery in these comparisons were diverse, and information regarding renal recovery in AKI-D was scarce^13–15^. Furthermore, the association between economic status and renal recovery probability is difficult to be determined meaningfully because of the foundational differences in etiology of AKI, practice pattern of AKI treatment, and the health care infrastructure, not to mention the significant under-report of data in lower-income regions^14^.

In Taiwan, the medical services for the total 23 million residents are provided by about 47,000 physicians in around 400 hospitals and ten thousand clinics, and over 99% of residents are covered by a single National Health Insurance with meager self-pay rates^1^. The high availability of medical services and the high coverage rate of medical insurance throughout the country minimize the confounding effects of health care infrastructure and economic consideration for seeking medical aids, making Taiwan an optimal place to evaluate the influence of regional economic status on renal recovery. Therefore, we conducted the current study in Taiwan to prove the hypothesis that the regional economic status is associated with the probability of renal recovery of critically ill patients with AKI-D.

## Materials and methods

### Study design and participants

This nationwide prospective multicenter study was conducted using the database of the nationwide epidemiology and prognosis of dialysis-requiring acute kidney injury (NEP-AKI-D) study^16,17^. Briefly, the NEP-AKI-D study was designed to enroll critically ill adult patients with AKI-D receiving RRT in the ICUs of both tertiary medical centers and regional hospitals located in the four geographical regions (north, middle, south, and east) of Taiwan. The four seasonal sampled months for enrolling participants were October 2014, along with January, April, and July 2015. The exclusion criteria included patients less than 20 years of age, patients with previous chronic dialysis before the index hospitalization, and patients who died within 48 h after admission to ICUs. After the enrollment process, we followed the clinical courses and documented the outcomes events of these enrolled participants until death or 90 days following hospital discharge. Then we evaluated the association between the regional economic status and the probability of renal recovery of these patients.

### Demographic and clinical covariates

The demographic information and clinical data were documented in the registry. The laboratory data and clinical variables, including severity scores at four-time points (hospital admission, ICU admission, RRT initiation, and hospital discharge), were recorded. (The details of demographic and clinical covariates were provided in Supplementary files).

Patients' disease severities were assessed using the Charlson Comorbidity Index (CCI), the acute physiology and chronic health evaluation II (APACHE-II) score, the sequential organ failure assessment (SOFA), and inotropic equivalent (IE)^18^. Sepsis was diagnosed according to the "sepsis-3" clinical criteria, which include (1) suspected or documented infection, along with (2) evidence of organ dysfunction represented by an acute increase in the SOFA score of ≥ 2 points^19^.

### Definitions and details about AKI and RRT

The AKI was diagnosed according to the Kidney Disease: Improving Global Outcomes (KDIGO) clinical practice guideline for AKI^20^. The definitions regarding the AKI and RRT were detailed in our previous work^16^ and the supplementary file (Supplementary files).

### Regional economic status

The regional economic status was defined by the "annual disposable income per capita (ADIPC)" of individual cities where the enrolled hospitals located within the period from July 1, 2014, to June 30, 2015. The per-capita expenditures were defined as the total individual expenditures divided by the total resident numbers of the city. The ADIPC was obtained from the "National Statistics, Republic of China (Taiwan)" website of "Directorate-General of Budget, accounting, and statistics, Executive Yuan, Taiwan" (accessed online: https://statdb.dgbas.gov.tw/pxweb/Dialog/CityItemlist_n.asp).

### Outcomes

The primary endpoint of the current study was renal recovery before death or 90 days following hospital discharge. We also additionally compared the all-cause mortality on the 90th day after hospital discharge. Renal recovery was defined as weaning from RRT for at least 7 days.

### Statistical analysis

The R 2.12.1 (R Foundation for Statistical Computing, Vienna, Austria) and Scientific Package for Social Science (PASW Statistics for Windows, Version 22.0, Chicago: SPSS Inc) software were applied for statistical analyses. A two-sided *p* ≤ 0.05 was considered statistically significant. Categorical variables were expressed as "numbers (percentages)." Continuous variables were expressed as mean ± standard deviation (SD) for those with normal distribution or median [interquartile range] for those with non-normal distribution. The chi-square test, or Fisher's exact test for those with an expected value of ≤ 5 in any box, was used to analyze categorical variables. The independent t-test for normal distribution, or the Mann–Whitney U test for non-normal distribution, was used to compare continuous variables. We used a multilevel discrete-time event history analysis and adopted a generalized additive model (GAM), with adjustment to risk factors, to evaluate the threshold of economic status. The model incorporated the subject-specific (longitudinal) random effects and expressed as the logarithm of the odds (logit)^21,22^. We demonstrated the association between the economic variable and the probability of renal function recovery. We further transformed the relevant economic variable into a categorical variable using the best cut-points determined by the GAM plots. Because of the high mortality in critical patients with AKI-D, we used the Fine and Gray competing risk regression model with mortality taken as a competing risk factor to calculate the subdistribution hazard ratio (sHR) and 95% confidence interval (CI) for the probability of renal function recovery^23,24^. The Cox proportional method was used to compare the mortality risk between groups.

### Ethics, consent, and permissions

The study design conformed to the ethical guidelines, and the Helsinki Declaration revised in 2013. The study was approved by the National Research Program for Biopharmaceuticals (NRPB)-Institutional Review Board (IRB) (NRPB2014050014) and IRBs of all the participating hospitals. The above IRBs waived the need for informed consent because of the de-identified personal information.

## Results

**Figure 1:**
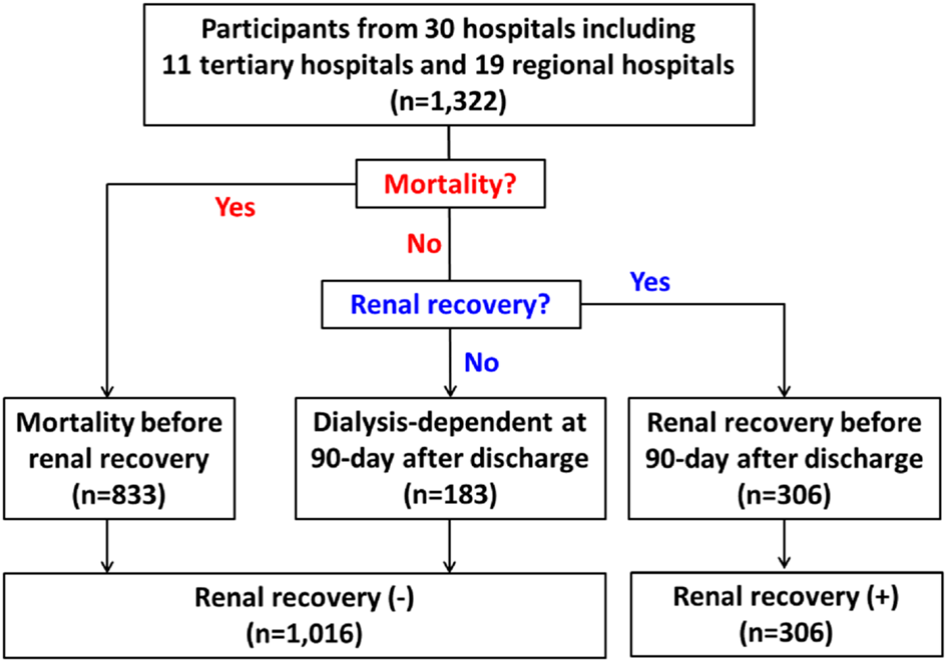
Flowchart of patient selection and categorization.

A total of 1,322 critically ill patients (67.1 ± 15.5 years of age, 36.2% female) with AKI-D in the ICUs were enrolled. Among them, 833 patients (63.1%) died, 306 patients (23.1%) had a renal recovery, and 183 patients (13.8%) were diagnosed with ESRD within the 90 days following discharge. The renal recovery rate was 23.1% in all patients and 62.6% in survivors within the 90 days. Overall speaking, 306 patients experience renal recovery, whereas 1,016 patients did not have renal recovery before death (in the non-survivors) or before the 90th day after discharge (in the survivors) (Fig. 1).

**Figure 2:**
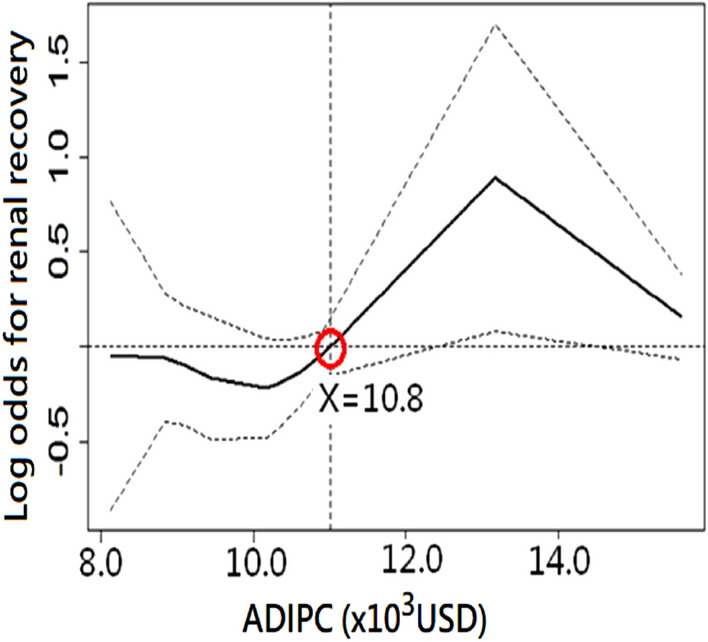
Generalized additive model plot demonstrating the association between economic status and the probability of renal recovery. *Note*: The model incorporated the subject-specific (longitudinal) random effects, expressed as the logarithm of the odds (logit). The probability of renal recovery was constructed with the ADIPC and was centered on having an average of zero over the range of the data. ADIPC, annual disposable income per capita; USD, United States dollar.

Next step, we demonstrated the association between ADIPC and the probability of renal recovery by the nonlinear GAM plot with adjustment to age, gender, baseline estimated glomerular filtration rate (eGFR), ventilator support, and SOFA at RRT initiation. From the plot, the best cut-point of ADIPC for differentiating the higher and lower probabilities of renal recovery was determined as 10.8 × 10^3^ United States dollars (Fig. 2).

### Clinical characteristics of the two groups stratified by economic status

According to the best cut-point of ADIPC, we categorized all patients into high (n = 992) and low economic status groups (n = 330). Compared to the low economic status group, the patients in the high economic status group had higher proportions of coronary artery disease (28.7% versus 22.4%, *p* = 0.03) and congestive heart failure (denoted as congestive heart failure with New York Heart Association Functional Classifications 3 and 4) (43.5% versus 20.0%). At hospital admission, the high economic status group had higher baseline CCI (7.0 ± 3.1 versus 6.3 ± 3.1 points, *p* = 0.01), BUN (59.1 ± 45.3 versus 49.5 ± 38.8 mg/dL), SCr (3.7 ± 3.4 versus 3.2 ± 2.9 mg/dL, *p* = 0.01) but lower IE (5.7 ± 14.8 versus 9.0 ± 21.4 points, *p* = 0.01) than the low economic status group (all *p* < 0.001 unless otherwise denoted).

At ICU admission, the high economic status group had a higher proportion of ventilator support (77.0% versus 50.9%) as well as higher BUN (66.4 ± 46.9 versus 57.1 ± 43.9 mg/dL, *p* = 0.01) and SCr (4.0 ± 3.3 versus 3.6 ± 3.0 mg/dL, *p* = 0.04), but lower surgical indication (21.3% versus 28.2%, *p* = 0.01) and IE (9.8 ± 17.3 versus 14.5 ± 30.0 points, *p* = 0.01). At RRT initiation, the high economic status group had higher BUN (88.5 ± 49.4 versus 81.8 ± 49.0 mg/dL, *p* = 0.03), potassium (4.7 ± 1.2 versus 4.5 ± 1.2 mg/dL, *p* = 0.03), and higher proportion of receiving diuretics (65.1% versus 58.8%, *p* = 0.04), but lower IE (11.7 ± 18.5 versus 19.0 ± 31.6 points) and SOFA scores (11.8 ± 4.2 versus 12.7 ± 4.4 points, *p* = 0.01) (all *p* < 0.001 unless otherwise denoted) (Tables 1 and S1, and Figure S1).

**Table 1:** Comparisons of essential or statistically different clinical variables between groups with high and low economic status.

	High economic status group (n = 992)	Low economic status group (n = 330)	*p* value
**Demographics**			
Gender, female	364 (36.7%)	115 (34.8%)	0.55
Age, years	67.4 ± 15.5	66.1 ± 15.5	0.18
Baseline eGFR, ml/min/1.73 m^2^	53.2 ± 48.5	58.4 ± 42.3	0.09
Diabetes mellitus	515 (51.9%)	168 (50.9%)	0.80
Coronary artery disease	285 (28.7%)	74 (22.4%)	0.03
Congestive heart failure	432 (43.5%)	66 (20.0%)	< 0.001
**Tertiary medical centers**	629 (63.4%)	218 (66.1%)	0.39
**At hospital admission**			
Charlson comorbidity index, points	7.0 ± 3.1	6.3 ± 3.1	0.01
BUN, mg/dL	59.1 ± 45.3	49.5 ± 38.8	< 0.001
SCr, mg/dL	3.7 ± 3.4	3.2 ± 2.9	0.01
IE, points	5.7 ± 14.8	9.0 ± 21.4	0.01
**At ICU admission**			
Surgical indication	211 (21.3%)	93 (28.2%)	0.01
Ventilator support	228 (77.0%)	168 (50.9%)	< 0.001
BUN, mg/dL	66.4 ± 46.9	57.1 ± 43.9	0.01
SCr, mg/dL	4.0 ± 3.3	3.6 ± 3.0	0.04
IE, points	9.8 ± 17.3	14.5 ± 30.0	0.01
**At RRT initiation**			
Heart rate, /minute	100.0 ± 23.7	100.7 ± 21.2	0.62
Respiratory rate, /minute	21.6 ± 6.7	21.1 ± 6.4	0.21
MAP, mmHg	79.8 ± 20.1	80.7 ± 18.8	0.47
Urine output, ml/day	551.1 ± 764.5	549.5 ± 728.8	0.97
PaO_2_/FiO_2_, mmHg	285.6 ± 205.5	287.0 ± 185.0	0.92
BUN, mg/dL	88.5 ± 49.4	81.8 ± 49.0	0.03
SCr, mg/dL	5.1 ± 3.1	4.9 ± 2.9	0.17
Potassium, mEq/L	4.7 ± 1.2	4.5 ± 1.2	0.03
Hemoglobin, g/dL	9.6 ± 2.3	9.6 ± 2.2	0.76
GCS, points	8.6 ± 4.3	7.9 ± 4.0	0.01
IE, points	11.7 ± 18.5	19.0 ± 31.6	< 0.001
APACHE-II, points	23.7 ± 7.2	23.9 ± 6.6	0.68
SOFA score, points	11.8 ± 4.2	12.7 ± 4.4	0.01
Diuretics	646 (65.1%)	194 (58.8%)	0.04
**Etiology of AKI**			
Shock	566 (57.1%)	217 (65.8%)	0.01
Sepsis	717 (72.3%)	217 (65.8%)	0.03
Nephrotoxic drug	51 (5.1%)	21 (6.4%)	0.40
Contrast media	64 (6.5%)	29 (8.8%)	0.17
**Indication of RRT**			
Azotemia	550 (55.4%)	164 (49.7%)	0.07
Fluid overload	560 (56.5%)	185 (56.1%)	0.95
Electrolyte imbalance	382 (38.5%)	116 (35.2%)	0.29
Oliguria	633 (63.8%)	236 (71.5%)	0.01
Acid-base imbalance	488 (49.2%)	161 (48.8%)	0.90

All the continuous variables were with normal distribution, expressed as mean ± standard deviation, and compared using the independent t-test. Categorical variables were expressed as case number (percentage) and compared using the chi-square test.

Congestive heart failure was denoted as congestive heart failure with New York Heart Association Functional Classifications 3 and 4.

AKI, acute kidney injury; APACHE, acute physiology and chronic health evaluation; BUN, blood urea nitrogen; eGFR, estimated glomerular filtration rate; FiO_2_, fraction of inspiration O_2_; GCS, Glasgow coma scale; ICU, intensive care unit; IE, inotropic equivalent; MAP, mean arterial pressure; PaO_2_ arterial partial pressure of O_2_; RRT, renal replacement therapy; SCr, serum creatinine; SOFA, sequential organ failure assessment.

### Clinical characteristics of the two groups stratified by renal recovery status

Compared to those without renal recovery, the patients with renal recovery were younger (64.4 ± 16.0 versus 67.9 ± 15.2 years) and had a lower proportion of congestive heart failure (32.0% versus 39.4%, *p* = 0.02). At hospital admission, the patients with renal recovery had lower baseline CCI (5.9 ± 3.0 versus 7.1 ± 3.1 points) but higher urine output (921.3 ± 1,099.1 versus 758.8 ± 828.3 ml/day, *p* = 0.02) than those without renal recovery. At ICU admission, the patients with recovery had a lower proportion of ventilator support (62.7% versus 72.8%, *p* = 0.01). They also had higher urine output (879.7 ± 985.8 versus 699.7 ± 819.6 ml/day, *p* = 0.01) and Glasgow coma scale (GCS) (10.3 ± 4.6 versus 9.4 ± 4.6 points, *p* = 0.01), but lower APACHE-II (21.4 ± 7.2 versus 22.8 ± 7.4 points, *p* = 0.01) and SOFA scores (9.6 ± 3.9 versus 10.4 ± 4.1 points, *p* = 0.01) than those without recovery (all *p* < 0.001 unless otherwise denoted).

**Figure 3:**
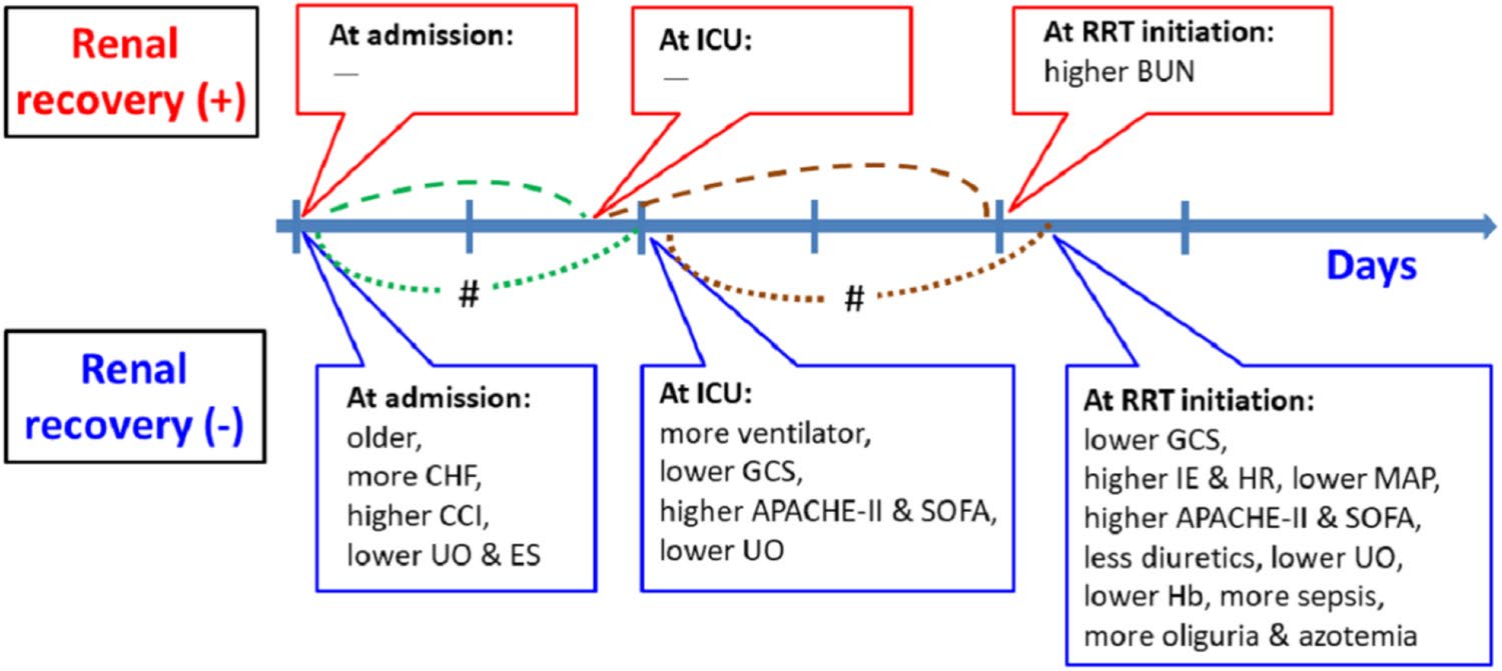
Time chart demonstrating the statistical differences between patients with and without renal recovery. *Note*: We only listed the variables which were statistically different between the two groups and were indicative of "worse condition" (ex: lower urine output) rather than "better condition" (ex: higher urine output). #Statistical significance of the corresponding period (lines with the same colors) between the two groups. APACHE, acute physiology and chronic health evaluation; BUN, blood urea nitrogen; CCI, Charlson Comorbidity Index; CHF, congestive heart failure; ES, economic status; GCS, Glasgow coma scale; Hb, hemoglobin; HR, heart rate; ICU, intensive care unit; IE, inotropic equivalent; MAP, mean arterial pressure; RRT, renal replacement therapy; SOFA, sequential organ failure assessment; UO, urine output.

At RRT initiation, the patients experiencing renal recovery had better hemodynamic status (higher mean arterial pressure [MAP] along with lower IE and heart rate [HR]), lower disease severity (higher GCS along with lower APACHE-II and SOFA scores), and better residual kidney function (higher urine output and lower BUN) than those without recovery. The patients with renal recovery had less proportion of "sepsis" (58.8% versus 74.3%) but more "nephrotoxic drug" (8.5% versus 4.5%, *p* = 0.01) as "etiology of AKI," as well as less azotemia (43.8% versus 57.1%) and oliguria (51.3% versus 70.1%) as "indications of RRT" than those without renal recovery. As to the economic status, the patients with renal recovery had high ADIPC (11.6 ± 2.0 versus 11.3 ± 1.9 × 10^3^ united states dollar [USD], *p* = 0.02) than those without recovery (all *p* < 0.001 unless otherwise denoted) (Fig. 3 and Table S2).

### Independent predictors for renal recovery

Finally, we conducted three models of competing risk regression analyses to evaluate the predictive ability of economic status for renal recovery. Model 1 included economic status alone. Model 2 included basic clinical variables besides economic status. Model 3 included clinical variables at RRT initiation in addition to the variables in Model 2. All the variables put into the models were of statistical significance in univariate analyses or those considered essential. We found that high economic status was independently associated with renal recovery in all three models of competing risk regression analyses (Table 2).

**Table 2:** Independent predictors of renal recovery within 90 days after discharge.

	Model 1			Model 2			Model 3		
Variables	sHR	95% CI	*p* value	sHR	95% CI	*p* value	sHR	95% CI	*p* value
Economic status, high versus low	1.335	1.056–1.689	0.016	1.334	1.054–1.688	0.017	1.422	1.022–1.977	0.037
Age, years^1^	–	–	–	0.989	0.982–0.996	0.002	0.991	0.983–0.999	0.042
Baseline eGFR, ml/min/1.73m^2^ ^1^	–	–	–	–	–	–	1.005	1.002–1.009	0.010
SOFA score, points^1,2^	–	–	–	–	–	–	0.917	0.874–0.962	< 0.001
Azotemia (indication)^3^	–	–	–	–	–	–	0.527	0.340–0.818	0.012
Oliguria (indication)^3^	–	–	–	–	–	–	0.721	0.528–0.984	0.036

^1^Every increment of one unit. ^2^At RRT initiation. ^3^With versus without. The analysis was performed using the Fine and Gray competing risk regression model with mortality taken as a competing risk factor.

The variable(s) put into the model:

**Model 1**: economic status (high versus low) only.

**Model 2**: economic status (high versus low), gender, age, baseline estimated glomerular filtration rate, congestive heart failure.

**Model 3**: economic status (high versus low), gender, age, baseline estimated glomerular filtration rate, congestive heart failure, variables at RRT initiation (heart rate, mean arterial pressure, urine output, Glasgow coma scale, blood urea nitrogen, inotropic equivalent, SOFA), sepsis (etiology), azotemia (indication), and oliguria (indication).

Congestive heart failure was denoted as congestive heart failure with New York Heart.

Association Functional Classifications 3 and 4.

sHR, subdistribution hazard ratio; CI, confidence interval; eGFR, estimated glomerular filtration rate; RRT, renal replacement therapy; SOFA, sequential organ failure assessment.

**Figure 4:**
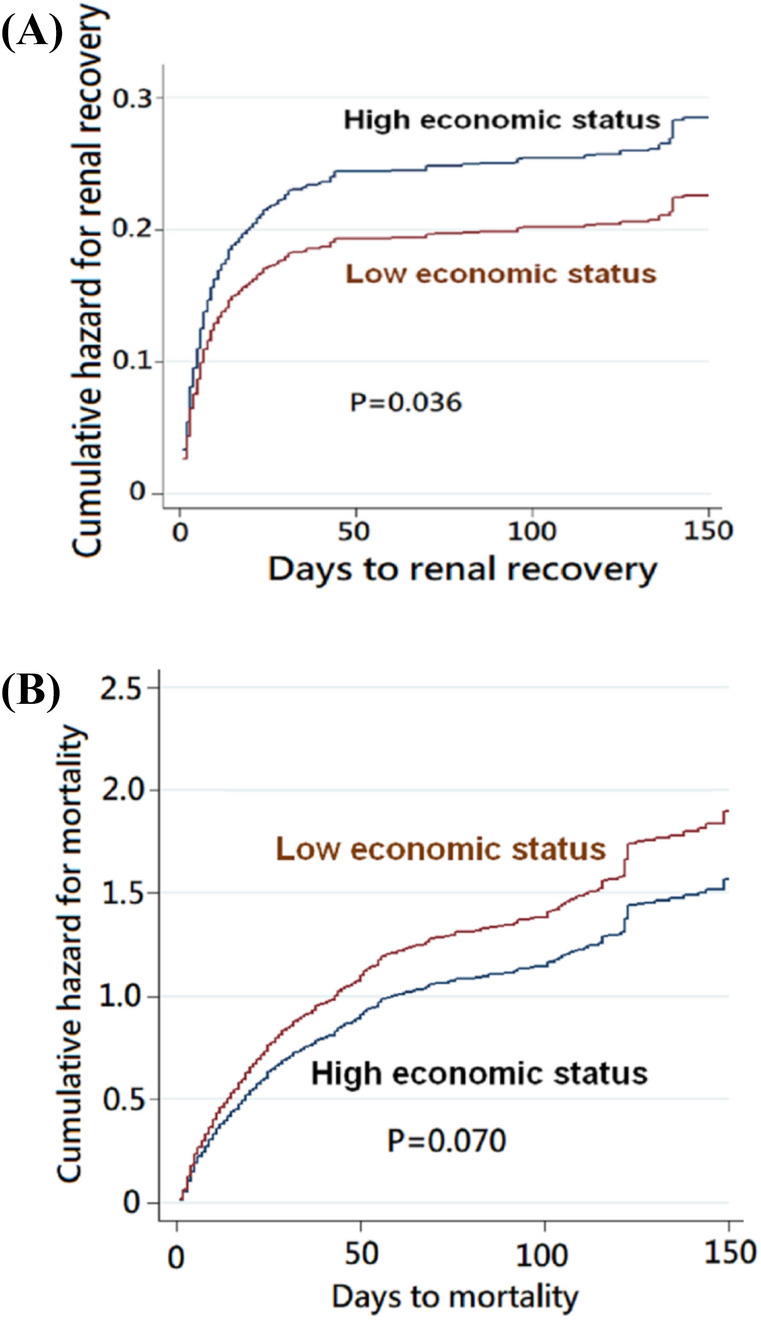
Cumulative hazards for (**A**) renal recovery and (**B**) mortality of groups with high and low economic status. *Note*: The plot (**A**) was drawn using the Fine and Gray competing risk regression model, with mortality taken as a competing risk factor. The plot (**B**) was drawn using the Cox proportional method.

Figure 4A, drawn by a competing risk regression model with mortality taken as a competing risk factor, showed that the high economic status group had a significantly higher probability for renal recovery (*p* = 0.036). The sharp upticks in both curves on the 149th day were resulted from the three deaths on the day. The association between economic status and mortality risk was not significant in Fig. 4B depicted by the Cox proportional plot with multiple factors adjustment (*p* = 0.070). The sharp upticks in both curves on the 122nd day were resulted from the four deaths on that day.

The adjusted factors of these two plots included all the variables, except for economic status, in Model 3 (Table 2). They included gender, age, baseline eGFR, CHF, variables at RRT initiation (heart rate, MAP, urine output, BUN, IE, SOFA), sepsis (etiology), azotemia (indication), and oliguria (indication).

## Discussion

To the best of our knowledge, the current nationwide study is an unprecedented study that found that the hospital located in the cities with high average ADIPC, comparing to those in the city with lower value, independently associated with a higher probability of renal recovery among critically ill patients with AKI-D.

In Taiwan, there are both local hospitals and tertiary medical centers in most of the cities. In the current study, the proportions of patients admitted to tertiary medical centers were not different between groups with high and low regional economic status (Table 1). The high availability of medical facilities allows residents to search for appropriate medical aids within the same cities they live in. Thus the average economic status of the cities where the hospitals located might be considered as the regional economic status of the patients live in the same city.

Besides, the hospitals enrolled in the study are either public hospitals or religious hospitals. All the medical professional works following the universal practice guidelines and regulations of the Taiwan National Health Insurance, and get payment from the health insurance rather than the patient personally. Thus the confounding influences from different hospital levels and varied practice patterns could be minimized. The findings of the current study pointed out the importance of regional economic status and opened a new avenue for better acute kidney disease care with an issue of economic status^25^.

In the current study, the patients with renal recovery were younger and had fewer comorbidities (lower prevalence of CHF and lower CCI), and clinically better than the patients who did not experience renal recovery throughout the whole hospital course (Fig. 3 and Table S1). Besides, high economic status, higher baseline eGFR, younger age, and lower SOFA scores were independently associated with a higher probability of renal recovery. In contrast, those with azotemia and oliguria as indications of RRT were less likely to have renal recovery among critically ill patients with AKI-D (Table 2). Most of the independent predictors of renal function recovery, except for economic status, are well recognized in the previous work^14,26–32^.

Regional socioeconomic deprivation has long been considered as an indicator of poor health and life expectancy^33^. In the nephrology field, socioeconomic status is well identified as an essential factor influencing the clinical outcomes of patients with CKD and kidney transplantation^34,35^, as well as the incidence and severity of AKI and mortality of AKI patients^25^.

Three potential reasons were proposed to explain the association between economic status and patients' prognoses^25,36–39^. Firstly, previous investigations found that low economic status disproportionally associates with a lack of health literacy, which refers to the ability to access, understand, and apply health-related information^36^. Limited health literacy links to the development of long-term health problems, decreased use of preventative medicine, deficient ability to handle medications, and elevated mortality^37^. Secondly, low economic status is also associated with worse patient-related health beliefs and behavior (e.g., higher prevalence of smoking, alcohol, obesity, poor control of blood sugar and blood pressure, unhealthy diet, and inactivity), as well as inadequate access to medical service (such as a delayed presentation to medical service)^25^. Thirdly, previous studies disclosed that insufficient insurance coverage results in a lower post-AKI follow-up rate and a lower prescription rate of drugs, which may be beneficial for renal function, such as angiotensin-converting enzyme inhibitors and angiotensin II receptor blockers^38,39^. Indeed, insurance coverage is theoretically an essential issue regarding the association between economic status and renal recovery. Although most of the residents in Taiwan are covered by the National Health Insurance with minimal copayment from the patients, the influence of financial consideration on the behavior of seeking medical service could not be eliminated by the higher insurance coverage rate.

In the current nationwide cohort, the patients with the high regional economic status group were more likely to be medical patients with a higher baseline comorbidity burden, including a worse baseline kidney function. They were also more likely to have sepsis, receive diuretics for AKI, and started RRT for hyperkalemia. Despite this unfavorable baseline kidney function, the patients with high regional economic status were still associated with a similar risk of mortality and higher possibility of renal recovery when compared to their counterparts.

The patients in the low regional economic status group had worse hemodynamic status (higher IE) throughout the whole hospitalization and had higher SOFA scores and more shock at RRT initiation (Tables 1 and S1, and Figure S1). For the patients with low regional economic status, the worse initial hemodynamic status in those with less underlying comorbidity burden could be potentially interpreted as "the delayed medical access." It is worthy of mentioning that delayed medical access exists even in a country with high availability of medical service and coverage rate of medical insurance. These findings underscored that, besides insurance coverage, other factors such as health literacy, as well as patient-related health beliefs and behavior are also essential regarding patients' prognoses.

In light of the results of the current study, the population level-based economic status is a crucial element in acute kidney disease and post-AKI care. To eliminate all avoidable deaths from AKI worldwide by 2025, the 0by25 initiative from the International Society of Nephrology proposed the 5Rs (Risk assessment, Recognition, Response, Renal support, and Rehabilitation) as a core to optimize the management of AKI worldwide^40^. Our findings extendedly add values in 5R approaches for acute kidney disease and post-AKI care. Examples include that physicians are encouraged to assess the probability of renal recovery (Risk assessment), as well as identify AKI-D patients experiencing renal recovery (Recognition) and stop the dialysis at the earliest time when the patients have renal recovery (Response and Renal support). Subsequently, the patients who experience renal recovery should receive regular follow-up (Rehabilitation). We also raised the incremental efforts in education to improve patients' health literacy, as well as health beliefs and behavior, might be an effective way, together with an increase in medical insurance coverage, to improve the probability of renal recovery in AKI patients.

Several limitations of this study should be addressed. Firstly, in the current study, which focused on the dialysis-withdrawal probability among AKI-D patients, the "indication bias" regarding the indication for dialysis existed. Nevertheless, the similar mortality rates between the high and low economic status groups minimized the "healthy worker survivor bias" regarding the dialysis-withdrawal probability. Secondly, the current study enrolled a sampled population of critically ill patients with AKI-D, including 23.0% of surgical patients and 77.0% of medical patients. Thus the results may not serve as a representative sample of all patients with varied types of AKI throughout the world. Thirdly, kidney recovery following AKI is challenging to define because it involves both structural and functional levels in the cell to whole organ level^40^. In the current study, we defined renal recovery by "dialysis-withdrawal." Although the decision of "dialysis-withdrawal" was made according to the clinical judgment of the physicians, the decision-making is clinical driven and of valuable clinical relevance^41^. Fourthly, despite the comprehensive adjustment for potential confounders in the current study, some factors which may associate with renal recovery might still be not available. Fifthly, as a population-level based investigation, the "ecological fallacy" is an unavoidable concern. Since we used population-level based data to demonstrate the association with renal recovery, the results in the current study are appropriate for population-level based application, but not for person-level practice. Further study using individuals' economic data is warranted to confirm our results and investigate the factors behind our findings.

In conclusion, we found that high regional economic status was independently associated with a higher possibility of renal recovery among critically ill patients with AKI-D, although with a similar mortality rate. The delayed presentation was a primary potential reason for the worse renal recovery rates in patients with low regional economic status. Further studies are warranted to exam the findings and determine the exact role of the economic status for renal recovery among patients with AKI-D.

## Supplementary information

Supplementary information
