# Supplementary material for: Association between regional economic status and renal recovery of dialysis-requiring acute kidney injury among critically ill patients
Source: Sci Rep. 2020 Sep 3;10:14573. doi: 10.1038/s41598-020-71540-7 (PMC7471258; doi:10.1038/s41598-020-71540-7)
Supplement: Supplementary file 1 — Supplementary information [file 41598_2020_71540_MOESM1_ESM.docx]

**Association between regional economic status and renal recovery of dialysis-requiring acute kidney injury among critically ill patients**

Chih-Chung Shiao, MD, Yu-Hsing Chang, MD, Ya-Fei Yang, MD, PhD, En-Tzu Lin, MD, Heng-Chih Pan, MD, Chih-Hsiang Chang, MD, Chun-Te Huang, MD, Min-Tsung Kao, MD, Tzung-Fang Chuang, MD, Yung-Chang Chen, MD, Wei-Chih Kan, MD, Feng-Chi Kuo, MD, Te-Chuan Chen, MD, Yung-Ming Chen, MD, Chih-Jen Wu, MD, PhD, Hung-Hsiang Liou, MD, Kuo-Cheng Lu, MD, Vin-Cent Wu, MD, PhD, Tzong-Shinn Chu, MD, PhD, Mai-Szu Wu, MD, Kwan-Dun Wu, MD, PhD, Ji-Tseng Fang, MD, Chiu-Ching Huang, MD, PhD.

**Supplementary information:**

***Details of demographic and clinical covariates***

The variables documented in the registry included demographic information, personal history, and baseline comorbidities, indications for intensive care unit (ICU) admission, details of surgery or medical procedures, etiologies of acute kidney injury (AKI), indications, and modalities of renal replacement therapy (RRT), patients' prognoses including survival status and renal outcomes. The laboratory data and clinical variables, including severity scores at four-time points (hospital admission, ICU admission, RRT initiation, and hospital discharge), were recorded.

***The diagnosis and etiologies of AKI***

The diagnosis of AKI is established according to the Kidney Disease: Improving Global Outcomes (KDIGO) clinical practice guideline for AKI [^1^](#_ENREF_1). The "baseline serum creatinine (SCr)" was defined as: (1) the lowest SCr level in an outpatient department (OPD) setting within three months before the index admission; (2) the latest SCr value during OPD follow-up for patients without OPD follow-up within three months before the index admission; (3) the lowest SCr value before dialysis during the index admission for patients without known previous SCr level; and (4) "not available" for those who do not have previous SCr data and receive RRT upon admission. The "AKI peak" was defined as the highest SCr value during the admission before dialysis. Patients fulfilling the diagnosis of AKI are prospectively enrolled in the current study and followed until hospital discharge [^2^](#_ENREF_2).

The categories of AKI include shock, sepsis, drug-related nephropathy, pigment nephropathy, contrast nephropathy, acute interstitial nephritis, acute glomerulonephritis, acute vasculitis, hepatorenal syndrome, abdominal compartment syndrome, obstructive uropathy, etc.[^2^](#_ENREF_2).

***The indication for RRT initiation and discontinuation***

The predefined indications for RRT initiation at our institutions are as follows: (1) azotemia (blood urea nitrogen (BUN) > 80 mg/dl and SCr > 2 mg/dl) with uremic symptoms (encephalopathy, pericarditis, pleuritis); (2) oliguria (urine amount <100 mL/8 hr) or anuria refractory to diuretics; (3) fluid overload refractory to diuretics use with a central venous pressure > 12 mm Hg; (4) hyperkalemia (serum potassium >5.5 mmol/L) refractory to medical treatment; and (5) metabolic acidosis (a pH < 7.2 in arterial blood gas) [^3^](#_ENREF_3)^,^[^4^](#_ENREF_4). However, the decision to start RRT is subject to the clinical judgment of the physicians.

***Modalities and intensity of RRT***

Double lumen catheters are placed for temporary vascular access. The decision of RRT modality is made by combining the clinical judgment of the consulting nephrologist and the in-charge intensivists. Generally speaking, intermittent hemodialysis (IHD) is used in hemodynamically stable patients (sequential organ failure assessment (SOFA) Cardiovascular Score of 0-1 for >24 hours), while continuous venous-venous hemofiltration (CVVH) and sustained low-efficiency daily dialysis (SLEDD) are reserved for patients with hemodynamic instability (SOFA Cardiovascular Score of 3-4). Besides, slow continuous ultrafiltration (SCUF) and continuous sustained low-efficiency daily diafiltration (c-SLEDD*-f*) are used in a medical center. Patients whose clinical condition has altered are allowed to modify dialysis modalities after consulting the nephrologist based on a universal protocol [^5^](#_ENREF_5). The intensity of RRT is also subject to the clinical judgment of the intensivists.

**Table S1. Comparisons of demographic and clinical variables between groups with high and low economic status**

|  | **High economic status group**  **(n=992)** | **Low economic status group**  **(n=330)** | **p-value** |
| --- | --- | --- | --- |
| **Demographics** |  |  |  |
| Gender, female | 364 (36.7%) | 115 (34.8%) | 0.55 |
| BMI, kg/m2 | 24.5 ± 4.9 | 25.9 ± 22.8 | 0.27 |
| Age, years | 67.4 ± 15.5 | 66.1 ± 15.5 | 0.18 |
| Baseline eGFR, ml/min/1.73m2 | 53.2 ± 48.5 | 58.4 ± 42.3 | 0.09 |
| Diabetes mellitus | 515 (51.9%) | 168 (50.9%) | 0.80 |
| Hypertension | 622 (62.7%) | 197 (59.7%) | 0.36 |
| Coronary artery disease | 285 (28.7%) | 74 (22.4%) | 0.03 |
| PAOD | 46 (4.6%) | 10 (3.0%) | 0.27 |
| Congestive Heart failure | 432 (43.5%) | 66 (20.0%) | < 0.001 |
| **Tertiary medical centers** | 629 (63.4%) | 218 (66.1%) | 0.39 |
| **At hospital admission** |  |  |  |
| Charlson comorbidity index, points | 7.0 ± 3.1 | 6.3 ± 3.1 | 0.01 |
| Urine output, ml/day | 811.4 ± 897.1 | 759.3 ± 923.9 | 0.40 |
| BUN, mg/dL | 59.1 ± 45.3 | 49.5 ± 38.8 | < 0.001 |
| SCr, mg/dL | 3.7 ± 3.4 | 3.2 ± 2.9 | 0.01 |
| GCS, points | 11.8 ± 4.3 | 12.0 ± 4.2 | 0.54 |
| IE, points | 5.7 ± 14.8 | 9.0 ± 21.4 | 0.01 |
| APACHE-II, points | 20.2 ± 7.6 | 19.5 ± 7.4 | 0.20 |
| SOFA score, points | 8.6 ± 4.1 | 8.9 ± 4.2 | 0.35 |
| **At ICU admission** |  |  |  |
| Surgical indication | 211 (21.3%) | 93 (28.2%) | 0.01 |
| Ventilator support | 228 (77.0%) | 168 (50.9%) | < 0.001 |
| Urine output, ml/day | 745.6 ± 831.9 | 728.8 ± 954.9 | 0.76 |
| PaO2/FiO2, mmHg | 294.5 ± 228.5 | 284.6 ± 218.1 | 0.49 |
| BUN, mg/dL | 66.4 ± 46.9 | 57.1 ± 43.9 | 0.01 |
| SCr, mg/dL | 4.0 ± 3.3 | 3.6 ± 3.0 | 0.04 |
| GCS, points | 9.6 ± 4.6 | 9.5 ± 4.7 | 0.66 |
| IE, points | 9.8 ± 17.3 | 14.5 ± 30.0 | 0.01 |
| APACHE-II, points | 22.6 ± 7.5 | 22.2 ± 7.1 | 0.50 |
| SOFA, points | 10.1 ± 4.1 | 10.5 ± 4.1 | 0.10 |
| **At RRT initiation** |  |  |  |
| Heart rate, /minute | 100.0 ±23.7 | 100.7 ± 21.2 | 0.62 |
| Respiratory rate, /minute | 21.6 ± 6.7 | 21.1 ± 6.4 | 0.21 |
| MAP, mmHg | 79.8 ± 20.1 | 80.7 ± 18.8 | 0.47 |
| Urine output, ml/day | 551.1 ± 764.5 | 549.5 ± 728.8 | 0.97 |
| PaO2/ FiO2, mmHg | 285.6 ± 205.5 | 287.0 ± 185.0 | 0.92 |
| BUN, mg/dL | 88.5 ± 49.4 | 81.8 ± 49.0 | 0.03 |
| SCr, mg/dL | 5.1 ± 3.1 | 4.9 ± 2.9 | 0.17 |
| Potassium, mEq/L | 4.7 ± 1.2 | 4.5 ± 1.2 | 0.03 |
| Hemoglobin, g/dL | 9.6 ± 2.3 | 9.6 ± 2.2 | 0.76 |
| GCS, points | 8.6 ± 4.3 | 7.9 ± 4.0 | 0.01 |
| IE, points | 11.7 ± 18.5 | 19.0 ± 31.6 | < 0.001 |
| APACHE-II, points | 23.7 ± 7.2 | 23.9 ± 6.6 | 0.68 |
| SOFA scores, points | 11.8 ± 4.2 | 12.7 ± 4.4 | 0.01 |
| Diuretics | 646 (65.1%) | 194 (58.8%) | 0.04 |
| **Etiology of AKI** |  |  |  |
| Shock | 566 (57.1%) | 217 (65.8%) | 0.01 |
| Sepsis | 717 (72.3%) | 217 (65.8%) | 0.03 |
| Nephrotoxic drug | 51 (5.1%) | 21 (6.4%) | 0.40 |
| Contrast media | 64 (6.5%) | 29 (8.8%) | 0.17 |
| **Indication of RRT** |  |  |  |
| Azotemia | 550 (55.4%) | 164 (49.7%) | 0.07 |
| Fluid overload | 560 (56.5%) | 185 (56.1%) | 0.95 |
| Electrolyte imbalance | 382 (38.5%) | 116 (35.2%) | 0.29 |
| Oliguria | 633 (63.8%) | 236 (71.5%) | 0.01 |
| Acid-base imbalance | 488 (49.2%) | 161 (48.8%) | 0.90 |
| **Modality of RRT** |  |  | 0.09 |
| CVVH | 357 (36.0%) | 141 (42.7%) |  |
| IHD | 570 (57.5%) | 171 (51.8%) |  |
| SLEDD | 65 (6.6%) | 18 (5.5%) |  |
| **Length of period** |  |  |  |
| Admission to ICU, days | 0 [0, 5] | 0 [0, 4] | 0.57 |
| ICU to RRT, days | 2 [0, 5] | 2 [1, 6] | 0.20 |
| Admission to RRT, days | 4 [1, 14] | 4.5 [1, 13] | 0.40 |

**Note:** Continuous variables were expressed as mean ± standard deviation for those with normal distribution or median [interquartile range] for those with non-normal distribution. The independent t-test for normal distribution or Mann-Whitney U test for non-normal distribution was used to compare continuous variables. Categorical variables were expressed as case number (percentage) and compared using the chi-square test.

Congestive heart failure was denoted as congestive heart failure with New York Heart Association Functional Classifications 3 and 4.

**Abbreviations:** AKI, acute kidney injury; APACHE, acute physiology and chronic health evaluation; BMI, body mass index; BUN, blood urea nitrogen; CVVH, continuous venous-venous hemofiltration; eGFR, estimated glomerular filtration rate; FiO2, fraction of inspiration O2; GCS, Glasgow coma scale; ICU, intensive care unit; IE, inotropic equivalent; IHD, intermittent hemodialysis; MAP, mean arterial pressure; NTD, New Taiwan dollar; PAOD, peripheral arterial occlusive disease; PaO2 arterial partial pressure of O2; RRT, renal replacement therapy; SCr, serum creatinine; SLEDD, sustained low-efficiency daily dialysis; SOFA, sequential organ failure assessment.

**Table S2. Comparisons of demographic and clinical variables between patients with and without renal recovery**

|  | **Renal recovery (+) (n=306)** | **Renal recovery (-) (n=1,016)** | **p-value** |
| --- | --- | --- | --- |
| **Demographics** |  |  |  |
| Gender, female | 110 (35.9%) | 369 (36.3%) | 0.9 |
| BMI, kg/m2 | 25.4 ± 5.8 | 24.6 ± 13.6 | 0.33 |
| Age, years | 64.4 ± 16.0 | 67.9 ± 15.2 | < 0.001 |
| Baseline eGFR, ml/min/1.73m2 | 57.5 ± 43.3 | 53.6 ± 48.1 | 0.20 |
| Diabetes mellitus | 165 (53.9%) | 518 (51.0%) | 0.40 |
| Hypertension | 184 (63.4%) | 625 (61.5%) | 0.59 |
| Coronary artery disease; | 88 (28.8%) | 271 (26.7%) | 0.47 |
| PAOD | 8 (2.6%) | 48 (4.7%) | 0.14 |
| Congestive heart failure | 98 (32.0%) | 400 (39.4%) | 0.02 |
| **Tertiary hospitals** | 185 (60.5%) | 662 (65.2%) | 0.14 |
| **At hospital admission** |  |  |  |
| Charlson Comorbidity Index, points | 5.9 ± 3.0 | 7.1 ± 3.1 | < 0.001 |
| Urine output, ml/day | 921.3 ± 1099.1 | 758.8 ± 828.3 | 0.02 |
| BUN, mg/dL | 57.7 ± 45.5 | 56.4 ± 43.4 | 0.65 |
| SCr, mg/dL | 3.9 ± 3.2 | 3.5 ± 3.3 | 0.08 |
| GCS, points | 12.1 ± 4.1 | 11.8 ± 4.3 | 0.32 |
| IE, points | 7.1 ± 16.5 | 6.3 ± 16.9 | 0.47 |
| APACHE-II, points | 19.8 ± 7.2 | 20.1 ± 7.7 | 0.64 |
| SOFA, points | 8.5 ± 3.9 | 8.7 ± 4.2 | 0.66 |
| **At ICU admission** |  |  |  |
| Surgical indication | 81 (26.5%) | 223 (21.9%) | 0.10 |
| Ventilator support | 192 (62.7%) | 740 (72.8%) | 0.01 |
| Urine output, ml/day | 879.7 ± 985.8 | 699.7 ± 819.6 | 0.01 |
| PaO2/FiO2, mmHg | 294.3 ± 202.3 | 291.3 ± 232.7 | 0.84 |
| BUN, mg/dL | 61.9 ± 48.9 | 64.7 ± 45.6 | 0.36 |
| SCr, mg/dL | 4.1 ± 3.3 | 3.9 ± 3.2 | 0.28 |
| GCS, points | 10.3 ± 4.6 | 9.4 ± 4.6 | 0.01 |
| IE, points | 9.9 ± 17.0 | 11.3 ± 22.4 | 0.34 |
| APACHE-II, points | 21.4 ± 7.2 | 22.8 ± 7.4 | 0.01 |
| SOFA, points | 9.6 ± 3.9 | 10.4 ± 4.1 | 0.01 |
| **At RRT initiation** |  |  |  |
| Heart rate, /minute | 97.8 ± 24.2 | 100.9 ± 22.6 | 0.04 |
| Respiratory rate, /minute | 20.9 ± 5.7 | 21.7 ± 6.8 | 0.08 |
| MAP, mmHg | 83.4 ± 19.2 | 79.0 ± 19.8 | 0.01 |
| Urine output, ml/day | 792.8 ± 951.6 | 477.8 ± 669.1 | < 0.001 |
| PaO2/ FiO2, mmHg | 298.9 ± 191.9 | 282.1 ± 202.9 | 0.20 |
| BUN, mg/dL | 79.0 ± 51.3 | 89.2 ± 48.5 | 0.01 |
| SCr, mg/dL | 5.3 ± 3.2 | 5.0 ± 3.0 | 0.24 |
| Potassium, mEq/L | 4.7 ± 1.3 | 4.6 ± 1.2 | 0.17 |
| Hemoglobin, g/dL | 10.3 ± 2.7 | 9.4 ± 2.1 | < 0.001 |
| GCS, points | 9.5 ± 4.3 | 8.1 ± 4.1 | < 0.001 |
| IE, points | 10.7 ± 18.2 | 14.3 ± 23.8 | 0.01 |
| APACHE-II, points | 21.5 ± 6.7 | 24.4 ± 7.0 | < 0.001 |
| SOFA, points | 10.8 ± 4.1 | 12.4 ± 4.2 | < 0.001 |
| Diuretics | 174 (56.9%) | 666 (65.6%) | 0.01 |
| **Etiology of AKI** |  |  |  |
| Shock | 170 (55.6%). | 613 (60.3%) | 0.15 |
| Sepsis | 179 (58.8%) | 755 (74.3%) | < 0.001 |
| Nephrotoxic drug | 26 (8.5%) | 46 (4.5%) | 0.01 |
| Contrast media | 26 (8.5%) | 67 (6.6%) | 0.25 |
| **Indication of RRT** |  |  |  |
| Azotemia | 134 (43.8%) | 580 (57.1%) | < 0.001 |
| Fluid overload | 161 (52.6%) | 584 (57.5%) | 0.15 |
| Electrolyte imbalance | 126 (41.2%) | 372 (36.6%) | 0.16 |
| Oliguria | 157 (51.3%) | 712 (70.1%) | < 0.001 |
| Acid-base imbalance | 136 (44.4%) | 513 (50.5%) | 0.07 |
| **Modality of RRT** |  |  | 0.38 |
| CVVH | 111 (36.3%) | 387 (38.1%) |  |
| IHD | 180 (58.8%) | 561 (55.2%) |  |
| SLEDD | 15 (4.9%) | 68 (6.7%) |  |
| **Economic variables** |  |  |  |
| Annual disposable income per capita, x10^3^ USD | 11.6 ± 2.0 | 11.3 ± 1.9 | 0.02 |
| **Length of period** |  |  |  |
| Admission to ICU, days | 0 [0, 1] | 0 [0, 5] | 0.02 |
| ICU to RRT, days | 1 [0, 3] | 2 [0, 7] | 0.05 |
| Admission to RRT, days | 2 [1, 6] | 5 [1, 15] | < 0.001 |

**Note:** Continuous variables were expressed as mean ± standard deviation for those with normal distribution or median [interquartile range] for those with non-normal distribution. The independent t-test for normal distribution or Mann-Whitney U test for non-normal distribution was used to compare continuous variables. Categorical variables were expressed as case number (percentage) and compared using the chi-square test.

Congestive heart failure was denoted as congestive heart failure with New York Heart Association Functional Classifications 3 and 4.

**Abbreviations:** AKI, acute kidney injury; APACHE, acute physiology and chronic health evaluation; BMI, body mass index; BUN, blood urea nitrogen; CVVH, continuous venous-venous hemofiltration; eGFR, estimated glomerular filtration rate; FiO2, fraction of inspiration O2; GCS, Glasgow coma scale; ICU, intensive care unit; IE, inotropic equivalent; IHD, intermittent hemodialysis; MAP, mean arterial pressure; NTD, New Taiwan dollar; PAOD, peripheral arterial occlusive disease; PaO2, arterial partial pressure of O2; RRT, renal replacement therapy; SCr, serum creatinine; SLEDD, sustained low-efficiency daily dialysis; SOFA, sequential organ failure assessment; USD, united states dollar.

**Figure S1. Time chart demonstrating the differences between groups with high and low economic status**

**Note:** We only listed the variables which were statistically different between the two groups, and were indicative of "worse condition" (ex: lower urine output) rather than "better condition" (ex: higher urine output).

**Abbreviations:** BUN, blood urea nitrogen; CAD, coronary artery disease; CCI, Charlson comorbidity index; ES, economic status; GCS, Glasgow coma scale; CHF, congestive heart failure; ICU, intensive care unit; IE, inotropic equivalent; K, potassium; RRT, renal replacement therapy; SCr, serum creatinine; SOFA, sequential organ failure assessment.


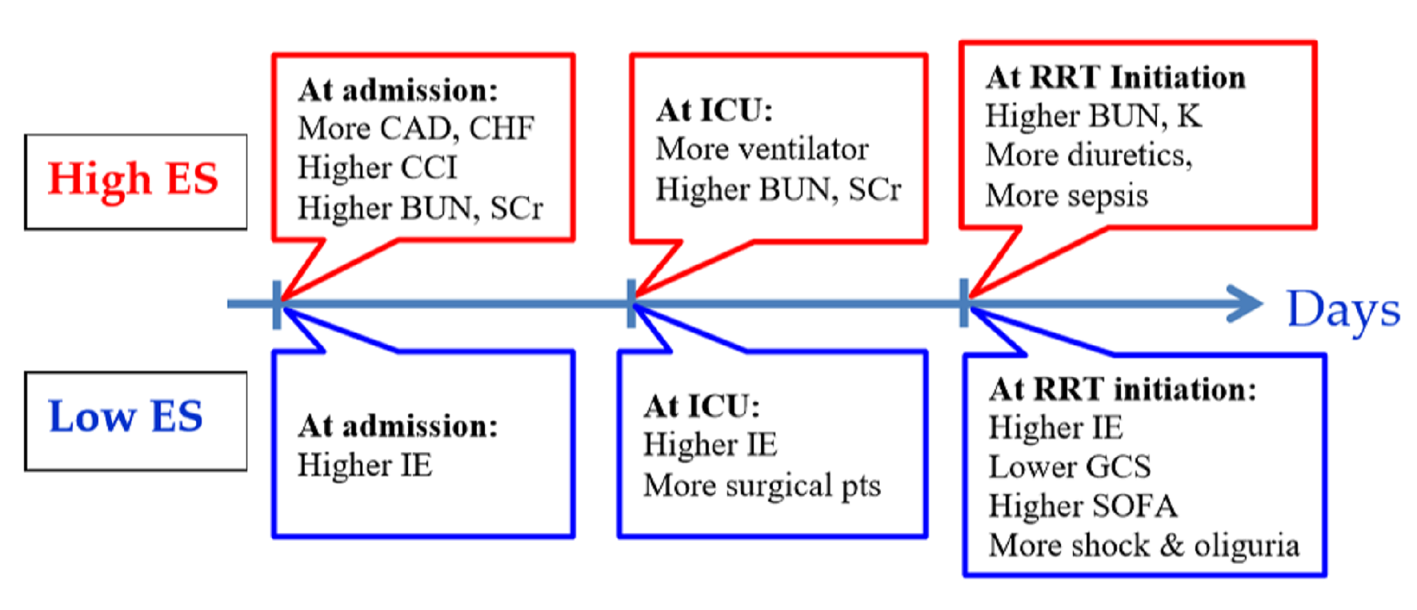


**References**

1 Section 2: AKI Definition. *Kidney international supplements* **2**, 19-36, doi:10.1038/kisup.2011.32 (2012).

2 Shiao, C. C. *et al.* Nationwide epidemiology and prognosis of dialysis-requiring acute kidney injury (NEP-AKI-D) study: Design and methods. *Nephrology (Carlton)* **21**, 758-764, doi:10.1111/nep.12670 (2016).

3 Wu, V. C. *et al.* Early renal replacement therapy in patients with postoperative acute liver failure associated with acute renal failure: effect on postoperative outcomes. *Journal of the American College of Surgeons* **205**, 266-276 (2007).

4 Lin, Y. F. *et al.* A modified sequential organ failure assessment score to predict hospital mortality of postoperative acute renal failure patients requiring renal replacement therapy. *Blood Purif* **26**, 547-554 (2008).

5 Palevsky, P. M., O'Connor, T., Zhang, J. H., Star, R. A. & Smith, M. W. Design of the VA/NIH Acute Renal Failure Trial Network (ATN) Study: intensive versus conventional renal support in acute renal failure. *Clin Trials* **2**, 423-435 (2005).
